# Stepped care for depression at integrated chronic care centers (IC3) in Malawi: study protocol for a stepped-wedge cluster randomized controlled trial

**DOI:** 10.1186/s13063-021-05601-1

**Published:** 2021-09-16

**Authors:** Ryan K. McBain, Owen Mwale, Todd Ruderman, Waste Kayira, Emilia Connolly, Mark Chalamanda, Chiyembekezo Kachimanga, Brown David Khongo, Jesse Wilson, Emily Wroe, Giuseppe Raviola, Stephanie Smith, Sarah Coleman, Ksakrad Kelly, Amruta Houde, Mahlet G. Tebeka, Samuel Watson, Kazione Kulisewa, Michael Udedi, Glenn Wagner

**Affiliations:** 1 RAND Corporation, Boston, 20 Park Plz, Boston, MA 02116 USA; 2 Partners In Health, Boston, MA USA; 3 Partners in Health, Neno District, Malawi; 4 Division of Hospital Medicine, Cincinnati Children’s Hospital Medical Center, Cincinnati, OH USA; 5 Division of Pediatrics, University of Cincinnati College of Medicine, Cincinnati, OH USA; 6 Brigham & Women’s Hospital, Boston, MA USA; 7 Harvard Medical School, Boston, MA USA; 8 RAND Corporation, Santa Monica, CA USA; 9 University of Birmingham, Birmingham, UK; 10 Blantyre College of Medicine, Blantyre, Malawi; 11 Ministry of Health, Lilongwe, Malawi

**Keywords:** Depression, Malawi, Randomized controlled trial, Problem Management Plus, Antidepressant therapy, Fluoxetine, Care integration, Stepped care, Chronic care, Low-resource setting

## Abstract

**Background:**

Malawi is a low-income country in sub-Saharan Africa that has limited resources to address a significant burden of disease—including HIV/AIDS. Additionally, depression is a leading cause of disability in the country but largely remains undiagnosed and untreated. The lack of cost-effective, scalable solutions is a fundamental barrier to expanding depression treatment. Against this backdrop, one major success has been the scale-up of a network of more than 700 HIV clinics, with over half a million patients enrolled in antiretroviral therapy (ART). As a chronic care system with dedicated human resources and infrastructure, this presents a strategic platform for integrating depression care and responds to a robust evidence base outlining the bi-directionality of depression and HIV outcomes.

**Methods:**

We will evaluate a stepped model of depression care that combines group-based Problem Management Plus (group PM+) with antidepressant therapy (ADT) for 420 adults with moderate/severe depression in Neno District, Malawi, as measured by the Patient Health Questionnaire-9 (PHQ-9) and Mini-International Neuropsychiatric Interview (MINI). Roll-out will follow a stepped-wedge cluster randomized design in which 14 health facilities are randomized to implement the model in five steps over a 15-month period. Primary outcomes (depression symptoms, functional impairment, and overall health) and secondary outcomes (e.g., HIV: viral load, ART adherence; diabetes: A1C levels, treatment adherence; hypertension: systolic blood pressure, treatment adherence) will be measured every 3 months through 12-month follow-up. We will also evaluate the model’s cost-effectiveness, quantified as an incremental cost-effectiveness ratio (ICER) compared to baseline chronic care services in the absence of the intervention model.

**Discussion:**

This study will conduct a stepped-wedge cluster randomized trial to compare the effects of an evidence-based depression care model versus usual care on depression symptom remediation as well as physical health outcomes for chronic care conditions. If determined to be cost-effective, this study will provide a model for integrating depression care into HIV clinics in additional districts of Malawi and other low-resource settings with high HIV prevalence.

**Trial registration:**

ClinicalTrials.govNCT04777006. Registered on 1 March, 2021

## Background

In low- and middle-income countries (LAMICs), mental health conditions like major depression often account for a larger burden of disease than HIV and malaria combined [1]; yet three-quarters of affected individuals receive no treatment [2]. The funding landscape accounts for much of this discrepancy: In 2017, international funding for HIV was $US 9.5 billion, compared to $US 130M for mental health conditions—roughly a 70-fold difference [3]. The economic impact of this under-investment, in terms of lost human capital, is expected to reach $30 trillion worldwide over a 20-year period [4]. Malawi represents a paradigm case: despite being the second poorest country in the world [5] and having one of the ten highest HIV incidences [6], collective efforts by the Ministry of Health and international donors have successfully built a system of care to address the HIV epidemic. Nevertheless, over 90% of those requiring treatment for depression have yet to receive care [7], even though depression is one of the leading causes of disability in Malawi [8]. One recent study estimated the point prevalence of depression in Malawi at 19% [9].

Underlying the paucity of depression care is a perception that, relative to treatments for infectious disease, treatments for mental health conditions are more time-intensive and less cost-effective [10]. However, research to date has two major shortcomings: First, evaluations often study the cost of stand-alone programs, rather than leveraging existing infrastructure to integrate care, which would reduce costs [11]. The HIV care platform in Malawi, comprising a network of community- and facility-based care, represents one such example [12]. Second, past interventions have underestimated total benefits of depression care, making treatment appear less cost-effective. For example, our past work has shown that depression treatment can lead to physical health improvements for comorbid conditions such as HIV through improved medication adherence [13, 14]. These indirect benefits should be quantified. Likewise, we have found household-level benefits, such as reduced need for emotional and financial supports [15], that have been overlooked [16–18].

Integrating depression care into the national HIV platform has the potential to represent a cost-effective, scalable solution in Malawi, consistent with best practices. Over the past 10 years, Malawi has enrolled over a million individuals in antiretroviral therapy. While many have passed away, current enrollment levels stand at 580,000 [19]. Over this same timeframe, HIV has evolved from an acute condition with poor prognosis, to a chronic condition with vastly improved survival rates [20]. Given this context, the HIV system is a strategic point of entry for screening and treating non-communicable diseases (NCDs) such as depression, as it represents a leverageable chronic care system that manages patients at high risk of mental health conditions [21]. In Neno District, HIV facilities have already transitioned into an integrated chronic care (IC3) platform for treating NCDs such as diabetes, hypertension, asthma, and epilepsy [22–24]. Likewise, community health screenings, routine in Malawi [25], could be leveraged to screen for NCDs such as depression.

We plan to evaluate an evidence-based stepped-care model of depression treatment integrated into Neno District’s IC3 system, assessing health outcomes and cost-effectiveness, inclusive of direct and indirect benefits. Specifically, the depression care model will recommend behavioral therapy for those with moderate depression, and a combination of behavioral therapy and pharmacological therapy for those with severe depression—aligned with Malawi’s aspirational guidance for mental health [26]. The behavioral therapy, group-based Problem Management Plus (PM+), has been recently developed by the World Health Organization (WHO) for delivery by lay health workers in resource-limited settings such as Malawi [27]. Preliminary evidence indicates substantial effects on reducing depression symptoms [28]. The antidepressant therapy protocol, in accordance with international guidelines, will utilize fluoxetine as a first-line treatment and amitriptyline as second line. Ultimately, this framework is intended to apply best practices in a delivery format that places significant consideration on the existing physical and human resources infrastructure available in rural Malawi. Moreover, while these represent evidence-based treatments, their implementation and effectiveness within a real-world low-resource setting such as Neno have not been studied.

Using a stepped-wedge cluster randomized trial (RCT), we will evaluate the effects of this stepped-care depression model, the protocol of which this paper describes in detail. The primary objective of the study is to assess whether the integrated depression care model is superior to usual care on (1) reducing depression symptoms and functional impairment (primary outcomes); (2) improving physical health for those with HIV, diabetes, and/or hypertension (secondary outcomes); and (3) reducing perceived mental health stigma and increasing retention in IC3 care (care processes). We will also evaluate the incremental cost-effectiveness of integrating evidence-based depression care, relative to usual care, and track mental health-related outcomes among household members of patients. If efficacious and cost-effective, this study will provide a model for integrating depression care into HIV clinics in Malawi, and the study will promote social wellbeing among household members.

## Methods

### Study design

**Fig. 1:**
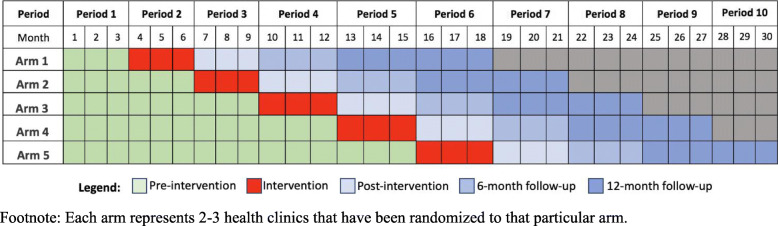
Stepped-wedge design of IC3D clinical trial. Each arm represents 2–3 health clinics that have been randomized to that particular arm

This is a prospective, stepped-wedge cluster randomized trial to evaluate the effects of an evidence-based depression care model, relative to usual care, on mental and physical health outcomes of patients seeking care at integrated chronic care centers (IC3) in Neno, Malawi. The study will take place at fourteen health clinics in Neno District, which will constitute our clusters. These fourteen clinics were selected because they represent all health facilities in Neno District, where the clinical trial will be taking place. Clinics will be randomized to one of five arms, each comprising 2–3 health clinics, such that that the clinics receive the intervention in random order and all clinics receive the intervention by the end of the study (Fig. 1).

A schedule of trial activities is shown in Table 1. Within each cluster, we will use an open cohort design of individual participants who screen positive for potential depression. All attendees at each clinic will be screened at each time point and screen-positive patients will enter the cohort and be followed for the rest of the study—including after initiation of depression care at their site to assess how depression and depression alleviation relate to our primary (depression symptom alleviation; alleviation of functional impairment) and secondary (HIV patients: viral load; hypertension patients: systolic blood pressure; diabetes patients: A1C or blood glucose levels; all patients: health utility index) outcomes, as well as processes of care (treatment uptake; adherence; perceived mental health stigma). Furthermore, prior to receipt of depression care, as well as 6 months later, one household member per participant will be interviewed in order to examine changes in their mental and physical health status. A cost-effectiveness analysis will be used to examine the incremental cost of integrated depression care (IC3D) over and above usual care within the IC3 framework.

**Table 1:** Schedule of enrollment, interventions, and assessments

Activity	Study period																			
	Year 1				Year 2				Year 3				Year 4				Year 5			
	Q1	Q2	Q3	Q4	Q1	Q2	Q3	Q4	Q1	Q2	Q3	Q4	Q1	Q2	Q3	Q4	Q1	Q2	Q3	Q4
Preparation																				
Develop measures	x	x																		
Develop protocols		x	x	x																
Hire/train staff				x																
Allocation																				
Clinic assignment				x																
Enrollment																				
Eligibility screen					x	x														
Informed consent					x	x														
Intervention																				
Depression care							x	x	x	x	x	x								
Usual care						x	x	x	x	x	x									
Assessments																				
Baseline					x	x	x													
Follow-up						x	x	x	x	x	x	x	x	x	x	x				
Analysis																				
Effectiveness																	x	x		
Cost-effectiveness			x	x															x	x

*Q* one quarter of a calendar year

### Randomization, allocation, and blinding

Health clinics will be randomly allocated to the five arms, the roll-out of which is spaced apart at 3-month intervals, with one exception: 13 potential combinations will be restricted from this algorithm, due to logistical considerations in the geographical layout of Neno District (note that over a quadrillion theoretical permutations are retained). This randomization will be executed by the project principal investigators using a random sequence generator in Stata 15 [29].

There is no way to blind the participants on whether or not they have received the intervention; this could potentially influence the outcomes, as clients may feel more or less incentivized to report feeling well in light of whether or not they have received the intervention. We do not see a way to prevent this potential bias, nor a way to distinguish such effects from actual intervention effects, but this limitation will be cited in reports of study findings. However, we will ensure that data analysts are blinded as to participant assignment, and those conducting interviews with patients on a three-month basis will also be blinded as to the enrollment status of participants. We do not foresee any circumstances under which unblinding would occur.

### Study setting

The study will take place at fourteen health facilities and five outreach clinics in Neno District. Ten health facilities are run solely by the Ministry of Health and the remaining four by the Christian Health Association of Malawi (CHAM) in collaboration with the Ministry of Health. Partners In Health—a health nonprofit organization—accompanies the Ministry of Health in the district. The project will receive technical implementation assistance from the RAND Corporation, Blantyre College of Medicine, Harvard Medical School, and University of Birmingham. Each of the participating health facilities provide HIV and NCD care and treatment, and range in size—from dispensaries that serve 50–250 patients per year to health centers serving roughly 250–500 patients per year to secondary-level hospitals providing care to over 1000 patients per year.

Patients in Neno District, Malawi, with chronic health conditions are enrolled and treated at integrated chronic care clinics (IC3)—an integration of Malawi’s national HIV system with NCD care—at regular intervals [30]. IC3 provides treatment for a broad array of chronic care conditions, including HIV, hypertension, diabetes, and epilepsy. The clinic is staffed by clinical officers, nurses, and support staff employed both by PIH and MOH. IC3 implementation assumes a modular framework, in which patients proceed station by station for screening and treatment administered by nurses, after which they receive a concluding consultation with a clinical officer. Supervision and mentorship are performed by district MOH coordinators and physicians along with support from PIH. In total, the IC3 model served approximately 13,000 patients with chronic care conditions in 2020.

A core group of staff for IC3 are based at the health facilities, while an additional group of staff travel to the health facilities 4–5 days a week to join these facility-based staff. This IC3 model leverages the existing HIV care and treatment model and is a solution for staffing shortages at smaller facilities given high volumes of patients with other acute needs.

### Study participants and eligibility criteria

**Fig. 2:**
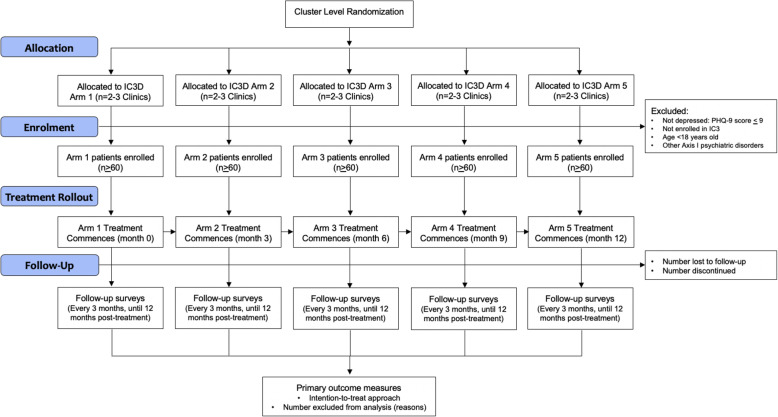
Trial flow chart

Initial eligibility criteria include (1) active patients at an IC3 in Neno District, and (2) age 18 or older. Given complexities associated with pediatric depression care, as well as age-related differences in the mode and content of behavioral and pharmacological interventions for depression among those in this age group, we are limiting the trial to adults [31] (Fig. 2).

**Fig. 3:**
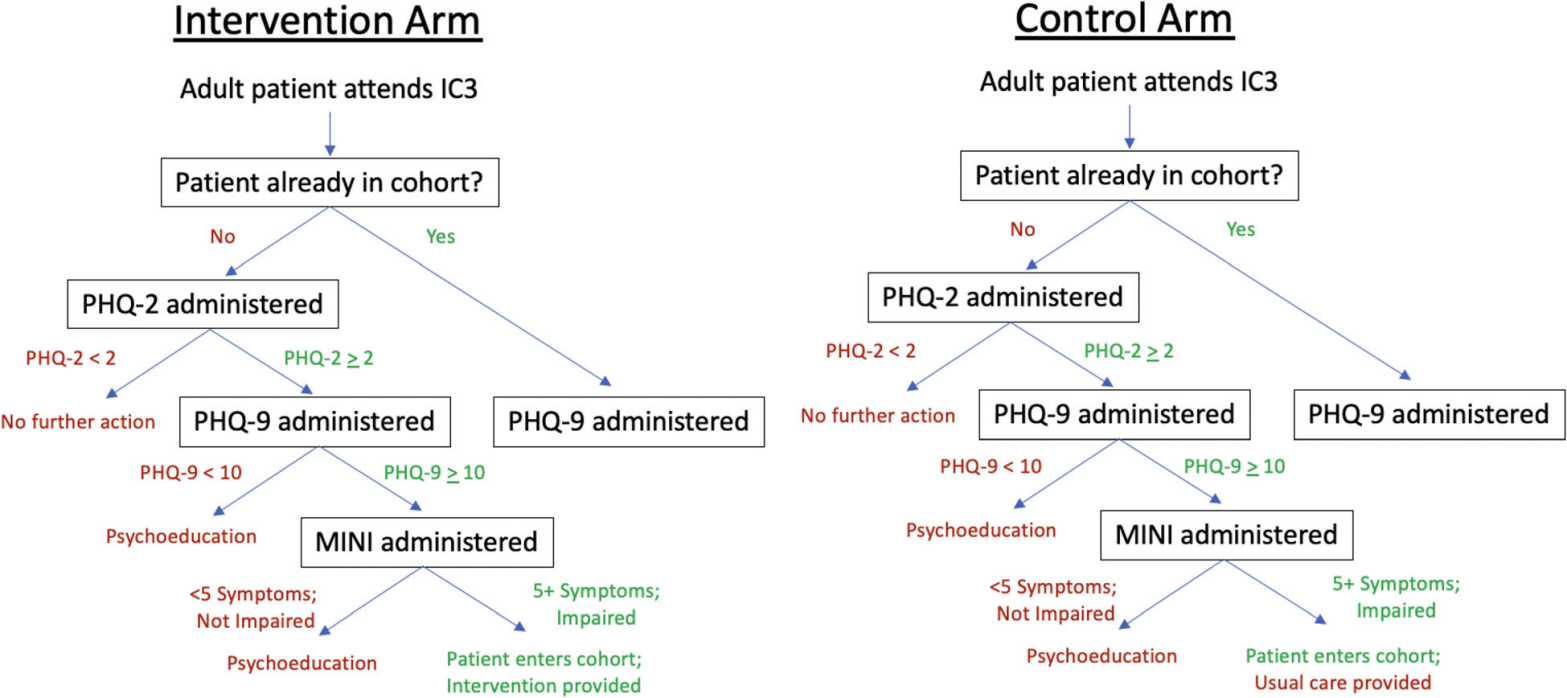
Care pathways for intervention and control arms

Screening, diagnosis, and enrollment will be administered by data clerks and counselors trained on the PHQ-2 and PHQ-9 [32], which have been locally validated in Malawi [33], as well as the Mini-International Neuropsychiatric Interview (MINI) [34], which will be administered by a counselor (see Fig. 3). The PHQ-2 assesses the frequency of depressed mood and anhedonia and has been implemented throughout Africa [35–37]. Administration of the PHQ-2 by data clerks as a first step has the potential to save time, given the number of attendees at IC3. Those who score 0 will not advance to the remaining PHQ-9 questions. Patients who screen positive with the PHQ-2 (score > 0; range 0–6) will immediately receive the remaining PHQ-9 questions from a counselor. A PHQ-9 score ≥ 10 (range, 0–27) represents a possible depressive disorder [32, 38]. Those with PHQ-9 < 10 (PHQ-9 score of 5-9 indicates mild depressive symptoms) will receive a brief, manualized psychoeducation session, and counselors will inform patients that screening will continue at IC3 on an ongoing basis. Those with PHQ ≥ 10 (moderate/severe depression) will proceed to receive the Mini-International Neuropsychiatric Interview (MINI) by counselors, on which 5 of 9 symptoms present and functional impairment constitute a diagnosis of depression. Analogous to the PHQ-9, those for whom a diagnosis of depression is not concluded will receive a brief, manualized psychoeducation session.

In the event that a patient proffers a non-zero response to the ninth question of the PHQ-9 (on suicidality), this will trigger initiation of a suicide risk assessment developed by the NIMH [39]. If the individual is identified as being at imminent risk of harm to him/herself or others, patients will be escorted to the supervising physician or another health provider at the health facility and initiate an interventional protocol.

### Treatment: usual care

In most of Malawi, including in Neno District, the usual care procedure for addressing depression is for patients who self-present with significant symptoms such as psychosis, suicidal ideation, or attempt or potential harm to others receive a referral either to a mental health clinical officer or nurse within the district (in Neno District there are three), or else to a mental health specialist at a regional hospital. Due to the limited supply of mental health clinicians in Neno District, those with comorbid conditions such as schizophrenia or more complex mood disorders such as bipolar disorder are prioritized for immediate intervention. Of those who presently receive treatment for overt mood disorders, treatment typically comprises pharmacotherapy—including fluoxetine or amitriptyline for depressive symptoms with individual counseling if time and staff are available. There is currently no system-wide model in IC3 or primary services for screening for depression. However, Neno District has begun screening and treatment for peripartum depression at one antenatal clinic—a system separate from the IC3 system which is the focus of this work. The inclusion of depression screening and psychoeducation, features of the control arm of this study, represent modest enhancements of the existing system from which the full IC3 model will be introduced.

With regard to IC3, services are condition-specific. To provide an example: HIV/AIDS is the most common chronic care condition treated at IC3, constituting approximately 70% of enrollees. Patients receiving antiretroviral therapy attend clinic every 3 to 6 months for evaluation (e.g., screening, adherence, viral load) and are provided with a necessary supply of antiretroviral medications until their scheduled return date. Patients with non-suppression, missed appointments, or poor adherence are evaluated on a monthly basis. Non-communicable disease (NCD) patients attend clinic every 3 months with 1-month appointments with poor disease control or missed appointments. They are screened for comorbid disease (e.g., HIV, tuberculosis [TB], other NCDs) and provided with the necessary supply of medications to their scheduled return date.

### Treatment: IC3D

The Integrated Chronic Care Clinics Depression Module (IC3D) draws from best evidence on collaborative care models for depression in low-resource settings [40, 41]. We will use a gold standard, stepped-care approach to offering psychological and pharmacologic treatment across all treatment facilities and clinics. Usual care will be augmented with evidence-based Problem Management Plus (PM+) through the provision of manualized, group-based counseling sessions, and antidepressant therapy (ADT) for those with severe depression (or if PM+ is declined), which is consistent with WHO mhGAP guidelines [42] for use of ADT. The primary components of the depression care model are described below.

#### Psychoeducation and treatment selection

If eligible for treatment, the counselor will inform the client of the availability of PM+ and ADT as treatment options, in addition to encouraging the client to access general supports—such as peer support groups—within Neno. Psychoeducation on depression, treatment course, possible side effects, and benefits of treatment will be provided. Clients will have the autonomy to select their preferred treatment, but the counselor will encourage clients with moderate depression (PHQ-9 < 15) to consider group-based PM+ counseling given its established efficacy and absence of significant side effect concerns, while adults with moderate-to-severe and severe depression (PHQ-9 ≥ 15) will be encouraged to consider joint ADT and group-based PM+. If the client remains depressed after 12 weeks of receiving either depression treatment modality (as measured by PHQ-9 at quarterly visits), s/he will be offered to switch to or add the other form of treatment.

In the event an enrolled individual no longer meets criteria at treatment initiation, the individual will leave the cohort and not be eligible for treatment but will continue to be evaluated every 3 months for future eligibility. Given that screening, diagnosis and enrollment will occur at all facilities over the full trial period, we expect any reduction in patients enrolled in the cohort due to lagged treatment initiation will be offset by newly identified individuals over this same period. This is consistent with an open cohort design for stepped-wedge trials [43].

#### Problem Management Plus (PM+)

Psychosocial counselors with a diploma from a Malawian university will receive a 1-week training course to administer group-based PM+ sessions, with ongoing mentorship and supportive supervision. PM+ is a cognitive-behavioral intervention that trains recipients to improve their management of practical problems and uses the term “problem management” rather than “problem solving” to emphasize that many problems encountered by individuals living in adverse circumstances may not be “solvable” [27]. The “plus” in PM+ underscores additional evidence-based behavioral strategies incorporated into the model, including stress management strategies, behavioral activation [44], and social support strengthening [45]. In total, PM+ comprises five sessions held once per week for 1.5. to 2.5 h per session. The model has shown success in reducing depression symptoms in low-resource settings such as Nepal [46] and Pakistan [28].

#### Antidepressant therapy (ADT)

We expect 20–25% of study participants—i.e., those who meet diagnostic criteria—to have severe depression and opt to enroll in ADT [47, 48]. Fluoxetine, a serotonin selective reuptake inhibitor (SSRI), and amitriptyline, a tricyclic antidepressant (TCA), are part of Malawi’s national formulary [49]. Neno District supply chain is supported by PIH, which reduces stock-outs relative to those observed in other Malawian settings. Potential side effects of ADT are modest but include nausea, insomnia, and nervousness [50] and will be communicated to participants prior to initiation. There is modest evidence ADT may increase the risk of suicidality [51]; this risk will be stated in informed consents, counselors will be trained to identify this risk, and patients will be formally assessed during outcome assessments, with the potential to trigger protocol for suicide assessment. Benefits of ADT often outweigh the risk of harm and negative impact of untreated depression. Fluoxetine is typically the first drug of choice because it is safer and better tolerated [52].

Daily dose will commence at 20 mg of fluoxetine, or 25 mg of amitriptyline, administered by trained clinical officers [49]. At monthly follow-up visits, a dose increment or medication change may be considered based on measures of treatment response and side effects, using an Antidepressant Side Effect Checklist—also administered by clinical officers [53]. This algorithm-based process will be repeated every other week until the patient is fully responding to treatment (PHQ-9 < 5) for a period of 3 months. Dose escalations of more than one increment and medication changes will be reviewed by a supervising mental health clinician with greater expertise.

#### Treatment monitoring

Schedule of follow-up visits to monitor side effects and treatment response will vary based on the treatment; for PM+, visits will be weekly for all five sessions, whereas for ADT, the first two visits will be every other week, and then the remainder will be monthly. A Depression Registry will be maintained by a project manager to record treatment data for each visit, which will facilitate future visits, supervision, and fidelity monitoring. Discontinuation will be considered if symptoms are in remission for 9 months [54]. If on ADT at end of study, it will be sustained as part of usual care.

#### Training, supervision, and fidelity

All counselors will be trained to implement the depression care model so that related burden is balanced and to prevent interruptions caused by staff changes. When possible, counselors will have received prior certification in psychosocial counseling from a local university; however, depending on recruitment success, other counselors will have no prior mental health training and served in a former capacity as a community health worker or similar position. Clinical officers responsible for administering ADT will have a degree in clinical medicine (mental health) from a university in Malawi (or equivalent) and will receive additional training on manualized ADT protocols prior to trial initiation [55].

Supervision will be conducted one-on-one with counselors and clinical officers—and provided by a lead clinician. Supervision will be weekly for the first month, then every other week for 3 months, and then monthly thereafter. Supervisors will be available 24/7 for suicide or emergency consultations. Regarding fidelity, supervisors will review charts of clients receiving ADT, and at each monthly supervision meeting assess adherence to the treatment protocol. For PM+, we will develop a checklist for the supervisors to use to rate fidelity to each component of the protocol for PM+.

### Primary outcomes

Measures will include surveys, laboratory assays, pharmacy data, and data abstracted from medical charts. Survey measures that have not been translated into Chichewa in the course of prior research will be translated using standard translation, back-translation methods, and will be administered at each assessment, unless otherwise noted.

#### Depression prevalence and severity

Depression symptoms will be measured with the PHQ-9 [38], which has been translated previously and locally validated [33]. We have elected to use PHQ-9 because it has been implemented throughout sub-Saharan Africa, including Malawi, where the scale has shown high sensitivity responding to treatment, as well as concurrent and predictive validity [7, 56–60]. It contains nine 4-point ordinal response items on topics such as hopelessness, psychosomatic response (e.g., sleeplessness, loss of appetite), and anhedonia. We will derive two outcome measures from this: (i) depression prevalence among patients attending the clinic and (ii) depression severity among those with depression. The numerator for the former is all those who screen positive as on the PHQ-9 (PHQ-9 ≥ 10), the denominator is all active adult patients at IC3. The latter measure will be represented by the PHQ-9 score. In both instances, the metric of interest was whether the prevalence/mean level of severity declines more rapidly among those in the treatment vs. comparison groups, as assessed at 3-month intervals.

#### Functional impairment

Functional impairment including aspects of daily functioning correspond to DSM diagnostic criteria [61]. We will evaluate this using the 12-item WHO Disability Assessment Schedule 2.0 (WHODAS) [62]. The WHODAS has been validated in a diverse range of sub-Saharan African contexts, including Malawi [63, 64], and covers topics like completing household chores, concentrating, and maintaining relationships. Akin to the depression measure, we will examine the comparative decline in mean functional impairment levels in the treatment vs. comparison groups, as assessed at 3-month intervals.

#### Overall health

A health profile will be generated for each individual enrolled in IC3D, using the EQ-5D-5L [65]—previously validated in Malawi [66]. This survey determines patients’ overall health at the time of interview and offers a cross-walk to quality-adjusted life years (QALYs) [67]. It is considered a continuous measure, and we will examine comparative improvements in overall health in the treatment vs. comparison groups, as assessed at 3-month intervals.

### Secondary outcomes

Secondary outcomes will constitute those measuring *indirect* benefits and costs of treatment. Indirect benefits will be incorporated into cost-effectiveness estimates by internalizing improvements among comorbid conditions such as HIV and hypertension, as well as reduced household burden of care. As with primary outcomes, we will examine comparative changes over time, in 3-month intervals, in the treatment vs. comparison groups. Continuous measures will examine change in mean scores at each interval, while dichotomous measures are evaluated with respect to prevalence rates.

#### ART adherence

Antiretroviral therapy (ART) adherence will be measured in terms of whether HIV+ enrollees have returned to an IC3 for antiretrovirals (ARVs) within the past 3 months or else defaulted, an approach considered more reliable than self-reports [68, 69]. This interval overlays with clinical protocols throughout Malawi in terms of the duration for which ARVs are supplied.

#### Viral load

Viral load will be measured as part of HIV+ patients’ clinical assessment pre-intervention, post-intervention, 6- and 12-month follow-up, consistent with ongoing care at IC3s. This is considered a key metric for determining treatment plans and is highly sensitive to compliance with prescribed ART regimens [70].

#### HIV disease staging

Staging will be evaluated every 3 months among HIV+ enrollees, consistent with IC3 visits. This approach to tracking disease progression is commonly used in low-resource settings [71].

#### Chronic care outcomes

Chronic care outcomes on hypertension, type-2 diabetes, and epilepsy will be abstracted from electronic medical records. Systolic blood pressure and adherence to antihypertensive medication (measured by adherence to clinic visits with medication dispersing) will be measured among those with hypertension. Among those with type-2 diabetes, adherence to quarterly visits with medication dispersement and A1C (or random blood sugar) levels will be abstracted. Among those with epilepsy, we will catalog the number of reported seizures in the past quarter, and adherence to antiepileptics as measured by quarterly medicine pickups.

#### Household burden of care

Lastly, household burden of care will be evaluated with household members using a locally adapted version of the Burden Assessment Schedule (BAS) [15], previously implemented by the team [17]. The survey assesses dimensions of emotional and functional burden of care, including missed work, guilt, and worry.

### Mediators and moderators

Our analyses will also include three mediators/moderators assessing potential pathways by which depression treatment leads to alleviation of depression symptoms, including enhanced social support, reduced HIV and depression-related stigma, and performance of psychosocial counselors as quantified by fidelity to PM+ protocols.

#### Perceived social support

Social support strengthening is a focus of PM+ [72]. This will be measured with the Multidimensional Scale of Perceived Social Support (MSPSS) [73], previously validated [74], to assess support before and after care.

#### Perceived stigma

Self-perceived stigma is associated with HIV status [75] and depression [76, 77], including in Malawi [78, 79]. We will adapt the AIDS-Related Stigma Scale (ARSS) and Internalizing Stigma of Mental Illness (ISMI) scale [80] in year 1 of the project—both designed for use in community-based settings in sub-Saharan Africa [81].

#### PM+ fidelity

Fidelity checklist scores will be generated for each counselor by a lead clinical officer or supervisor. Research has shown that fidelity protocols are predictive of patient outcomes in the context of depression care [82].

### Household interviews

Adult household members of participants—one per household—will be identified for participation-based referral by the study participant and/or self-identification as social support in close contact with the study participant. This individual will be informed that the research team is conducting a broad study on the relationship between individual and household health. Household interviews will chiefly comprise a subset of the battery administered to participants: specifically, the PHQ-9, WHODAS, and EQ-5D-5L (primary outcomes). In addition, household members will complete the BAS, as described above.

### Process evaluation

In the final year of the project, we will conduct qualitative interviews to assess provider and patient experiences on their perceived effects of the intervention, as well as implementation barriers and strategies used to solve them. The goal is to identify key lessons learned and to ready the intervention tools for scale-up at end of study.

#### Provider experience

In year 5, we will interview up to 10 providers including clinical officers who prescribed ADT, as well as psychosocial counselors who led PM+ sessions. In addition to discussing perceived strengths and weaknesses of the intervention and its implementation, we will also discuss the training and supervision process, as well as provider job satisfaction and burnout.

#### Patient experience

A random sample of 20 participants (10 male, 10 female) will be interviewed after their final study assessment to assess their experiences with the intervention—including logistical aspect of participation, impact on personal mental health and wellbeing, and as changes in relationships at the household level.

### Sample size

We assumed *n* = 30 enrollees per facility (total *n* = 420) during each 3 month “step”, based on an estimated 10% depression prevalence in Malawi [7, 83]. We further assumed loss to follow-up of 15%, based on intervention trials we have conducted in similar settings [11, 84]. To reduce loss to follow-up, Partners In Health dispatches community health workers to the households of any patients who have missed medical care visits for more than 2 weeks—which has proven highly successful in ensuring continuity of care.

The power calculation is based on the model described in the analysis section (see the following) and a two-sided null value of no effect [85]. To indicate the precision of the study design, we report “minimum detectable effect” sizes across the range of our primary outcomes at 80% power, with a type I error rate of 0.05, and conservative intraclass correlation coefficient (ICC) of 0.05, a cluster autocorrelation coefficient (CAC) of 0.80, and an individual autocorrelation coefficient (IAC) 0.80. Table 2 reports these effect sizes.

**Table 2:** Minimum detectable effect

Outcome	Baseline assumed	Denominator population	Treatment effect	Lower minimum detectable effect	Upper minimum detectable effect
Depression prevalence	10%	All active patients (*n* = 300 per cluster)	Odds ratio	0.97	1.03
Depression severity	N/A (SD = 1, standardized effect size)	All patients with depression (*n* = 30 per cluster)	Absolute effect	− 0.14	0.14
WHODAS	N/A (SD = 1, standardized effect size)	All patients with depression (*n* = 30 per cluster)	Absolute effect	− 0.14	0.14
EQ-5D-3L utility score	N/A (SD = 1, standardized effect size)	All patients with depression (*n* = 30 per cluster)	Absolute effect	− 0.14	0.14

### Analysis of intervention effects

The data from the trial will be analyzed using a generalized linear mixed model framework, which is standard for stepped-wedge cluster designs [86, 87]. The patients participating form an open cohort with repeated measures, as it will be possible to link each patient taking a PHQ-9 screening and any subsequent exams over time. The models will be at the patient level, nested within clinic-months and clinics. The primary outcomes are (i) prevalence of depression, (ii) depression severity (PHQ-9 score) conditional on having depression, (iii) WHODAS score conditional on having depression, and (iv) utility score (0 to 1) from the EQ-5D-5L. The first outcome is a dichotomous event for if the patient has depression or not, while the remaining outcomes are treated as continuous. For the principal analysis of the trial, we used the “complete” stepped-wedge of data from months 1 to 18; long-term follow-up will constitute a secondary analysis.

We specify the following model for patient *i* = 1, …, *N* in clinic *j* = 1, …, *J* at time period *t* = 1, …*T*:

$$ {y}_{ijt}\sim F\left(g\left({\eta}_{ijt}\right)\right) $$

$$ {\eta}_{ijt}=\mu +{x}_{ijt}^{\prime}\beta +{z}_{jt}^{\prime}\gamma +\delta {D}_{(i) jt}+{\theta}_i+{\alpha}_j+{\phi}_{jt}+{\tau}_t $$

where *y*_*ijt*_ is the outcome, *F*(.) is an appropriate likelihood and *g*(.) is a suitable link function, *x*_*ijt*_ and *z*_*jt*_ are respectively patient- and clinic-level covariates, *τ*_*t*_ are monthly fixed effects, and ${\theta}_i\sim N\left(0,{\sigma}_{\theta}^2\right)$, ${\alpha}_j\sim N\left(0,{\sigma}_{\alpha}^2\right)$, and ${\phi}_{jt}\sim N\left(0,{\sigma}_{\phi}^2\right)$ patient-, clinic-, and clinic-time random effects term. We will take both intention to treat (ITT) and per protocol approaches. For the ITT analysis, the variable *D*_*jt*_ is an indicator equal to one if cluster *j* is in the intervention state at time *t* and zero otherwise; thus, *δ* represents our estimated treatment effect. For per protocol analysis, *D*_*ijt*_ will be a patient-level variable equal to one if the clinic was in the intervention state and the patient completed the treatment as defined above, and zero otherwise. For dichotomous outcomes, we will use a Bernoulli distribution with logistic link function, for continuous outcomes a normal distribution with identity link function. The same models will be used for secondary outcomes. Missing data will be addressed using multiple imputation analysis with 10,000 imputation cycles.

All models will be estimated using restricted maximum likelihood using the R package lme4. We will report point estimates, confidence intervals, and *p*-values but not make any claims of “statistical significance” given recent strong arguments against doing so [88]. *P*-values will be based on the null hypotheses *H*_0_ : *δ* = 0 versus the two-sided alternatives *H*_1_ : *δ* ≠ 0 in each of the models. Given there are multiple primary outcomes, we will adjust reported *p*-values for multiple testing using a stepdown method, which provides an efficient means of controlling the family-wise error rate [89]. We will derive the exact distributions of the test statistics to perform the stepdown procedure using a (pseudo-)permutation test approach, by re-randomizing clusters to different sequences in the stepped-wedge design 100,000 times [90]. This method ensures appropriate control of the family-wise error rate and avoid potential biases resulting from small numbers of clusters.

### Cost and cost-effectiveness analysis

The research team will employ time-driven activity-based costing (TDABC) to gather program costs before (year 1) and after (year 4) intervention roll-out. TDABC is a gold standard, patient-centered approach: It begins by studying the flow of patients through the health system, and then measuring human and other resources expended. Baseline costs, gathered in Y1, will assess the cost of care as usual for individuals utilizing IC3s. A cycle of care will be annualized as four quarterly IC3 visits, inclusive of testing and labs, according to diagnosis. Itemized expenditures will be accrued from PIH’s financial software system [91]. To assess economic costs, such as subsidized ARVs by PEPFAR, we will use external cost sources such as management Science for Health’s International Medical Product Price Guide (MSH-IMPPG) [92]. As the cost comparator, we will replicate the cost exercise in year 4, when depression care is operating at scale, again tracing individuals according to service type—now including depression care.

Health measures will be converted to quality-adjusted life years (QALYs) using the EQ-5D-3L cross-walk between domain-specific outcomes [11]. QALY estimates will be related to base case and treatment cost estimates. For formal CEA, we will use a gold standard Markov chain Monte Carlo (MCMC) simulation-based approach [93] in TreeAge [94], from a societal perspective [95]. For sensitivity analyses, we will vary discount rates from 0-5%, based on health-adjusted life expectancy [96]. Unit of interest is the incremental cost-effectiveness ratio (ICER) of cost per QALY, comparing the cost of integrated chronic care before and after introduction of the depression treatment.

### Ethics

The study protocol has been reviewed and approved by the National Health and Science Research Council administered by the Government of Malawi, and by the RAND Corporation in Santa Monica, California. Any protocol modifications will be submitted to the IRB for review, and participants will be informed if warranted. The trial is registered with the NIH clinical trial registry (ClinicalTrials.gov) and assigned the number NCT04777006. All items from the WHO Trial Registration Data Set can be found within this protocol, as reflected on ClinicalTrials.gov.

We do not expect any medication-related adverse events beyond that of routine medical care and use of antidepressant therapy. All potential side effects will be outlined to patients during the informed consent process. Patients will be assessed and monitored with regards to psychiatric symptoms and treatment side effects by their provider on a standardized schedule using the Antidepressant Side Effect Checklist.

Given the potential severity of depression among those in the sample, some will express suicidal thoughts. Counselors will be trained to implement a standardized protocol when patients report suicidal thoughts, including assessment of the severity of the ideation, intent and means for carrying out any intent for suicide, and activation of a plan to keep the patient safe. In order to maintain the scientific integrity of this human subject research project, and to protect the safety of its research participants, we will assemble a Data Safety Monitoring Board (DSMB) comprising members with appropriate clinical and technical expertise. The DSMB will have the responsibility of assuring that participants are not being exposed to unnecessary or unreasonable risks as a result of the pursuit of the study’s scientific objectives. The study team also contains two clinical supervisors with advanced training, who will be responsible for clinical mentorship as well as weekly audits using structured supervision checklists to ensure fidelity to antidepressant and PM+ protocols.

No clinic-level data will be collected, apart from patient-level identifiers that allow us to determine the clinic at which patients received treatment. At the patient level, data will be collected through two modes: First, clinical officers will engage in routine clinical data entry into patient electronic medical records, stored on an encrypted server. Computers to access this server are password protected and require login from individual providers. Second, research data clerks and counselors will use CommCare—a mobile data collection platform—to interview patients every 3 months. Data will be entered into the password-protected tablet within the CommCare application, which itself requires a username and password to enter. Data will be uploaded from tablets to an encrypted server at the end of each workday.

### Availability of data

For dissemination purposes, reporting results will be documented on ClinicalTrials.gov in accordance with NIH requirements. Information submitted will occur no later than 12 months after the primary completion date. Results produced by this investigation will be presented at conferences where applicable and published in a timely fashion. In the final year of the project, we will also host a conference and invite government officials to discuss findings in the broader context of Malawian policy initiatives. All final peer-reviewed manuscripts that arise from this proposal will be submitted to PubMed Central for open access. When applicable, deidentified analytic code and the associated datasets will be deposited in public repositories—including Harvard University’s Dataverse database: https://dataverse.harvard.edu.

## Discussion

This study will conduct a district-wide stepped-wedge cluster randomized trial to assess the effects of an evidence-based stepped-care depression treatment model vs. usual care on depression symptom alleviation as well as quantifying improvements in physical health and household dynamics. While there is evidence of the deleterious effects of depression on mental health, this will be one of the first studies to quantify treatment benefits on physical health outcomes and household-level outcomes and to incorporate these knock-on effects in cost-effectiveness estimates. We anticipate that by leveraging the existing infrastructure of the robust HIV care delivery system, the IC3 model, and by quantifying these knock-on effects, expansion of depression care will be highly cost-effective and financially feasible for scale-up in Malawi.

Building on our research with task-shifted evidence-based depression care for HIV clients in Uganda and our use of problem-solving therapy (PST) and ADT in low-resource settings such as Rwanda and Haiti [97, 98], this study is specifically designed to test a group-based model of PM+ that WHO has advocated for broad scale-up. Moreover, the model focuses on an existing and growing cadre of counselors throughout Malawi to deliver group-based PM+ and will measure fidelity to protocol execution, among other measures. Through close partnership with the Ministry of Health, in the final year of this project, we intend to host a conference reviewing major findings of this work and to facilitate a discussion—including a technical expert panel—to outline potential strategies for wider scale-up.

## Data Availability

The authorship team will have access to the final trial dataset. All final peer-reviewed manuscripts that arise from this proposal will be submitted to PubMed Central for open access. When applicable, deidentified analytic code and the associated datasets will be deposited in public repositories—including Harvard University’s Dataverse database: https://dataverse.harvard.edu.

## Abbreviations

A1C: Hemoglobin A1C
ADT: Antidepressant therapy
ARSS: AIDS-related stigma scale
ART: Antiretroviral therapy
ARVs: Antiretroviruals
BAS: Burden assessment scale
CAC: Cluster autocorrelation coefficient
DSMB: Data and safety monitoring board
EQ-5D-5L: EuroQol 5 dimensions 5 levels
HIV: Human immunodeficiency virus
IAC: Individual autocorrelation coefficient
IC3: Integrated chronic care clinics
IC3D: Integrated Chronic Care Clinics Depression Module
ICC: Intraclass correlation coefficient
ICER: Incremental cost-effectiveness ratio
ISMI: Internalized stigma of mental illness scale
ITT: Intention to treat
LAMIC: Low- and middle-income county
MCMC: Markov chain Monte Carlo
MSPSS: Multidimensional scale of perceived social support
NCD: Non-communicable disease
NIMH: National Institute of Mental Health
PHQ-2: Patient Health Questionnaire-2-Item
PHQ-9: Patient Health Questionnaire-9-Item
PIH: Partners In Health
PM+: Problem Management Plus
QALY: Quality-adjusted life year
RCT: Randomized controlled trial
SSRI: Serotonin selective reuptake inhibitor
TB: Tuberculosis
TCA: Tricyclic antidepressant
TDABC: Time-driven activity-based costing
WHO: World Health Organization
WHODAS: World Health Organization Disability Assessment Schedule
